# Comparison of intraocular pressure-lowering effects of ripasudil hydrochloride hydrate for inflammatory and corticosteroid-induced ocular hypertension

**DOI:** 10.1371/journal.pone.0185305

**Published:** 2017-10-02

**Authors:** Mami Yasuda, Kei Takayama, Takayuki Kanda, Manzo Taguchi, Hideaki Someya, Masaru Takeuchi

**Affiliations:** Department of Ophthalmology, National Defense Medical College, Tokorozawa, Japan; Massachusetts Eye & Ear Infirmary, Harvard Medical School, UNITED STATES

## Abstract

Ocular hypertension (OHT) caused by inflammation or corticosteroid treatment is a common complication of uveitis. Ripasudil hydrochloride hydrate (K-115) is reportedly efficacious for lowering intraocular pressure (IOP). We retrospectively compared the IOP-lowering effect of K-115 for inflammatory and corticosteroid-induced OHT associated with uveitis. Thirty-six consecutive eyes of 27 patients with uveitis-associated OHT (20 and 16 eyes with inflammation- and corticosteroid-induced OHT, respectively) were treated with K-115 with or without other anti-glaucoma agents. In the inflammation-induced OHT, mean IOP and aqueous flare significantly decreased (P < 0.001 and P = 0.035, respectively), changing from 26.4 ± 7.5 mmHg and 28.1 ± 15.0 photon counts per millisecond (pc/ms) at the initial assessment to 17.9 ± 5.4 mmHg and 17.1 ± 10.7 pc/ms at the last visit, respectively. In the corticosteroid-induced OHT, mean IOP significantly decreased (P = 0.0005), changing from 26.7 ± 7.8 mmHg and 18.7 ± 11.2 pc/ms to 18.6 ± 8.8 mmHg and 22.6 ± 15.3 pc/ms, respectively; conversely, aqueous flare remained unchanged. In the inflammation-induced OHT, K-115 was more efficacious in the eyes with higher IOP. Neither remarkable adverse effects nor exacerbation of uveitis were observed in the eyes of either group during the observation period. K-115 decreased IOP in both inflammation- and corticosteroid-induced OHT associated with uveitis and played a synergistic role in reducing ocular inflammation in uveitis treatment.

## Introduction

Elevation of intraocular pressure (IOP) is a common complication in the management of uveitic patients and can occur in any type of uveitis and at any time during the course of the disease [1]. A variety of pathogenetic factors, including corticosteroid treatment, is involved in the development of ocular hypertension (OHT), which are classified as: inflammation-induced OHT and corticosteroid-induced OHT [1]. Anti-glaucoma agents, such as topical prostaglandin analogues, β-blockers and carbonic anhydrase inhibitors are often used to treat uveitis-associated OHT [2]. Because most uveitic patients receive topical corticosteroids and mydriatic agents for ocular inflammation, the anti-OHT agent prescribed is commonly used concurrently with anti-inflammatory agents [1, 2].

Rho-associated kinase (ROCK) regulates various cellular functions such as shape, motility, secretion, proliferation, gene expression, proliferation, and inflammation. ROCK signalling is activated by either secreted bioactive molecules or integrin activation following extracellular matrix binding [3]. This leads to the polymerisation of actin stress fibres and the formation of focal adhesion. Actin cytoskeleton modulation has been suggested as being involved in aqueous outflow regulation, and research has indicated that ROCK inhibitors reduce IOP and alter the cellular characteristics of the human trabecular meshwork [4].

The ROCK pathway inhibits carbachol-induced constriction of the ciliary muscle, suggesting effects on the ciliary body; further, decreased apoptosis of non-pigmented ciliary epithelial cells occurs in eyes with acute anterior uveitis. Ripasudil hydrochloride hydrate (K-115), a small-molecule ROCK inhibitor developed for the treatment of glaucoma and OHT [5], was approved for use in Japan in 2014. K-115 directly alters the extracellular matrix of the trabecular meshwork, cell shape and Schlemm’s canal [6, 7].

In this study, we investigated the efficacy and safety of K-115 on OHT associated with uveitis, and assess whether there were any differences in these effects between inflammation- and corticosteroid-induced OHT.

## Methods

### Patients

We retrospectively reviewed the clinical charts of patients with uveitis-associated OHT who first visited the National Defense Medical College Hospital between March 2015 and December 2016. The clinical diagnosis of uveitis was based on medical history and typical slit-lamp biomicroscopic and fundus findings. To assist with diagnosis, peripheral blood examination including angiotensin-converting enzyme and human leukocyte antigen analyses were performed. Uveitis was classified according to the recommendations proposed by the International Uveitis Study Group (IUSG), taking into consideration the latest recommendations [8, 9], as previously described [2]. OHT was diagnosed if an IOP of 21 mmHg or higher was recorded during at least two consecutive visits within 2 to 14 days. Exclusion criteria were corneal diseases, primary glaucoma, exfoliation syndrome or a history of trauma or surgery to the globe. Patients who had undergone small-incision cataract surgery more than 1 year ago were included.

Our retrospective procedures conformed to the tenets of the World Medical Association's Declaration of Helsinki. The National Defense Medical College Hospital Ethics Review Board approved this retrospective analysis of patient data. Written informed consent was obtained from all patients prior to accessing their medical record data in this research.

### Anti-inflammatory medications

Corticosteroid-induced OHT was defined as an IOP higher than 21 mmHg during corticosteroid administration, and as an IOP decrease after discontinuation or dose reduction of corticosteroids with concurrent administration of K-115 to control IOP [2]. Although classification into inflammation- or corticosteroid-induced OHT was difficult in several cases, the decisions of the individual uveitis specialists who treated those patients were adopted. For inflammation-induced OHT, topical or systemic corticosteroids were started or increased to resolve ocular inflammation when the fixed combination therapy was initiated. On the other hand, in corticosteroid-induced OHT, 0.1% dexamethasone was replaced to systemic corticosteroids in the active phase of uveitis, and 0.1% dexamethasone was discontinued or replaced to 0.1% fluorometholone in the remission phase. Although the use of systemic corticosteroids was considered a cause of OHT in two eyes of two patients with Vogt–Koyanagi–Harada disease and six eyes of three patients with scleritis, corticosteroid treatment or tapering was continued when K-115 was initiated.

### Examinations

Best-corrected visual acuity (BCVA) was measured at the initiation using a standard Japanese visual acuity chart. The decimal BCVA was converted to the logarithm of the minimum angle of resolution (logMAR) for statistical analysis, as previously described [10]. IOP was measured by Goldmann applanation tonometry and indirect ophthalmoscopy at the initiation and each subsequent visit. Anterior aqueous flare was measured five times with a Kowa FM-700 Aqueous Flare Meter (Kowa Medicals, Nagoya, Japan) in photon counts per millisecond (pc/ms), and the mean of five times was used in the analysis.

### Outcomes and statistical analysis

IOP and anterior aqueous flare at the initiation of K-115 and at the last visit during therapy were used in the analysis. The chi-square test with Fisher’s test was used to assess the differences between the inflammation- and corticosteroid-induced OHT groups. The Mann–Whitney U-test was used to assess the changes over time between the initial assessment and those at final visit. P-values less than 0.05 were considered statistically significant. Multiple linear regression analysis was used to evaluate the correlation between the changes of IOP/anterior aqueous flare and independent variables such as the type of OHT, age, sex, type of uveitis (granulomatous/non-granulomatous), prescribed medications (none, prostaglandin analogues, β-blockers, carbonic anhydrase inhibitors or α2-agonists) and IOP and anterior aqueous flare at the initiation.

## Results

### Patients’ characteristics

The characteristics of eyes with uveitis-associated OHT in this study are shown in Table 1.

**Table 1:** Patients’ characteristics in the inflammation- and corticosteroid-induced ocular hypertension groups.

		Inflammation	Corticosteroid	P
Number of eyes (people)		20 (16)	16 (11)	
Male/female (people)		5/11	6/5	
Mean age (years)		62.1 ± 13.2	63.0 ± 15.2	0.36*
Mean observation duration (months)		5.2 ± 3.0	5.2 ± 2.7	0.43*
Mean BCVA (logMAR)		0.47 ± 0.82	0.48 ± 0.80	0.44*
Diagnosis of uveitis (eyes)				
	Scleritis-associated uveitis	5	2	
	Behçet's disease		6	
	Herpes uveitis	3		
	Sarcoidosis	2	1	
	Vogt–Koyanagi–Harada disease		2	
	Cytomegalovirus-associated uveitis	1		
	Unknown	9	5	
Prescribed anti-glaucoma agents				
	Prostaglandin analogues	15	13	0.71^#^
	β-blockers	12	9	
	Carbonic anhydrase inhibitors	7	5	
	α2-agonists	5	8	

*: analysed by the Mann–Whitney U-test

^#^: analysed by the chi-square test with Fisher’s test; BCVA, best-corrected visual acuity

Twenty eyes of 16 patients with inflammation-induced OHT (inflammation group; male, five patients; mean age, 62.1 ± 13.2 years) and 16 eyes of 11 patients with corticosteroid-induced OHT (corticosteroid group; male, six patients; mean age, 63.0 ± 15.2 years) were included. The diagnoses and prescribed anti-glaucoma agents in the inflammation- and corticosteroid-induced groups were also shown in Table 1. There were no significant differences in all parameters between the groups. No severe adverse effects caused by K-115 were observed in any patients.

### IOP reduction and anterior aqueous flare changes in the two groups

**Fig 1:**
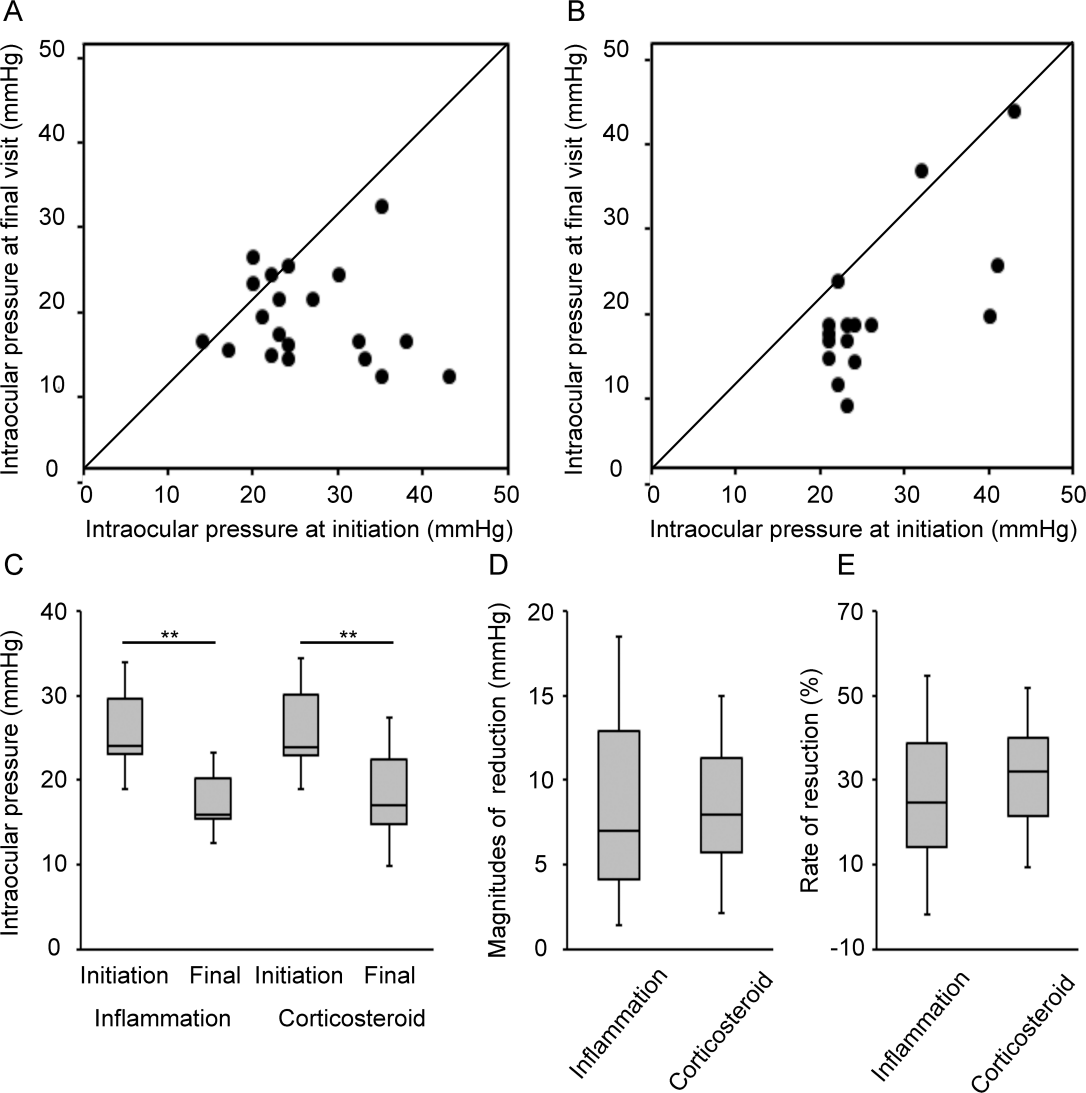
Changes in intraocular pressure in the inflammation- and corticosteroid-induced ocular hypertension groups. **(A)** IOP at the initiation and last visit in the inflammation group. **(B)** IOP at the initiation and last visit in the corticosteroid group. **(C)** The mean IOP at the initiation and last visit in the two groups. The mean IOP was reduced significantly by K-115 initiation in both groups. **(D)** The magnitudes of IOP reduction in the two groups. No significant differences were found between the two groups **(E)** The rates of IOP reduction in the two groups. No significant differences were found between the two groups. **: P < 0.01.

Fifteen (75%) of 20 eyes in the inflammation group and 14 (87.5%) of 16 eyes in the corticosteroid group showed a decrease in IOP from the initiation to the last visit (**Fig 1**A and **1**B).

The mean IOP was reduced significantly by K-115 initiation: from 26.4 ± 7.5 mmHg (95% CI, 23.1–29.7 mmHg) to 17.9 ± 5.4 mmHg (95% CI, 15.5–20.2 mmHg) in the inflammation group (P < 0.001) and from 26.7 ± 7.8 mmHg (95% CI, 23.3–30.1 mmHg) to 18.6 ± 8.8 mmHg (95% CI, 14.8–22.5 mmHg) in the corticosteroid group (P = 0.005) (**Fig 1**C). Post-treatment IOP did not differ between the two groups. The magnitudes of IOP reduction were 8.5 ± 10.0 mmHg (95% CI, 4.1–12.9 mmHg) in the inflammation group and 8.1 ± 6.5 mmHg (95% CI, 5.2–10.9 mmHg) in the corticosteroid group (**Fig 1**D), and the rates of IOP reduction were 26.5 ± 28.3% (95% CI, 14.1–38.9%) in the inflammation group and 30.8 ± 20.9% (95% CI, 21.7–40.0%) in the corticosteroid group (**Fig 1**E). No significant differences were found between the two groups.

**Fig 2:**
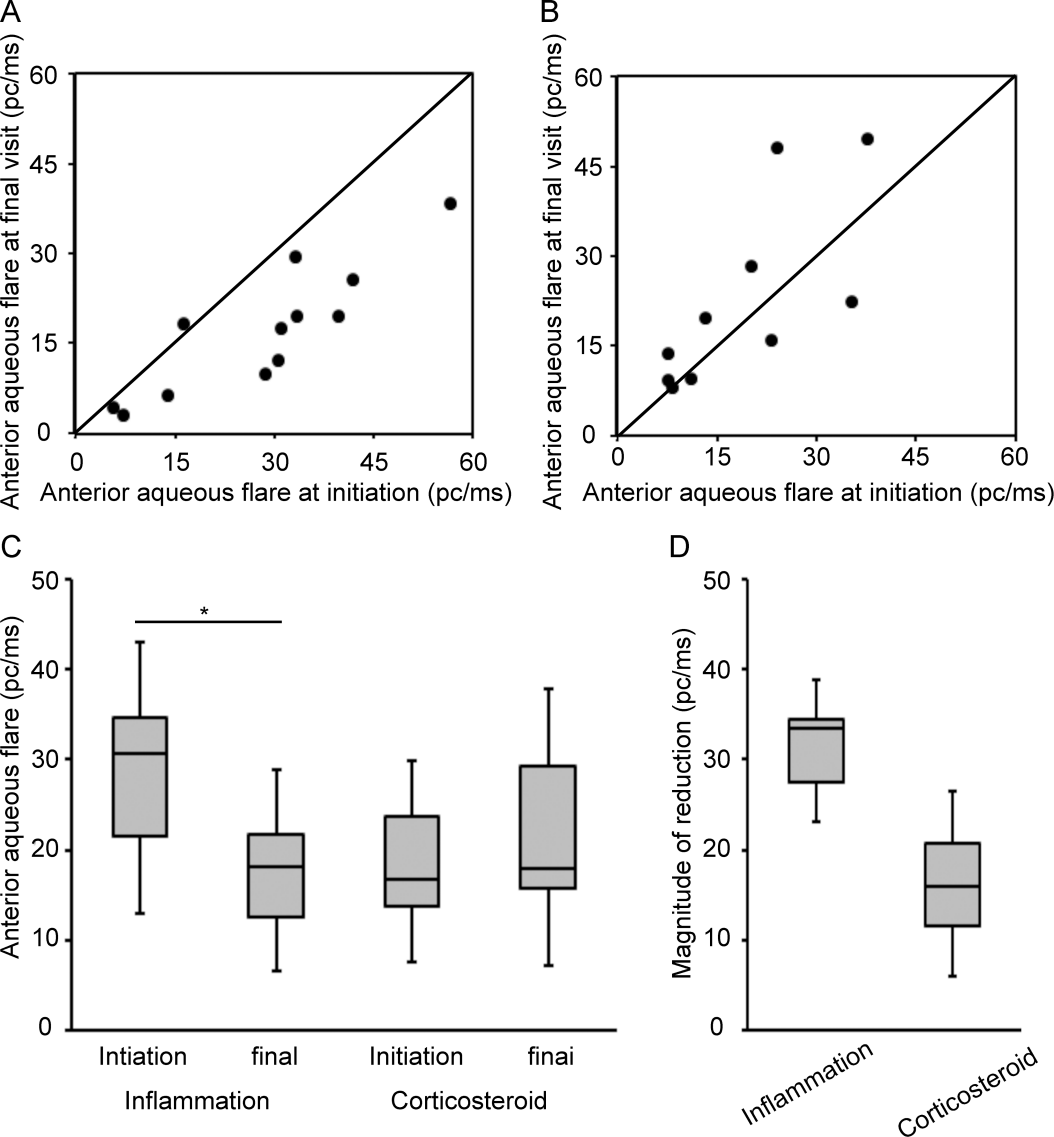
Changes in anterior aqueous flare in the inflammation- and corticosteroid-induced ocular hypertension groups. **(A)** Anterior aqueous flare at the initiation and last visit in the inflammation group. **(B)** Anterior aqueous flare at the initiation and last visit in the corticosteroid group. **(C)** The mean anterior aqueous flare in the two groups at the initiation and last visit. In the inflammation group, the mean anterior aqueous flare was significantly decreased. (**D)** The magnitude of change in anterior aqueous flare in the two groups. *: P< 0.05.

Next, we evaluated the changes in anterior aqueous flare in both groups. Eleven (75%) of 12 eyes in the inflammation group and four (40.0%) of 10 eyes in the corticosteroid group showed decreased anterior aqueous flare from the initiation to the last visit (Fig 2A and 2B).

In the inflammation group, the mean anterior aqueous flare was significantly decreased (P = 0.035), from 28.1 ± 15.0 pc/ms (95% CI, 21.5–34.7 pc/ms) to 17.1 ± 10.7 pc/ms (95% CI, 12.5–21.8 pc/ms); conversely, no significant change in anterior aqueous flare was observed in the corticosteroid group (Fig 2C; 18.7 ± 11.2 pc/ms [95% CI, 13.8–23.6 pc/ms] at the initiation and 22.6 ± 15.3 pc/ms [95% CI, 15.8–29.2 pc/ms] at the last visit). There was no significant difference between the two groups in the magnitude of anterior aqueous flare reduction (Fig 2D).

### Correlation and predictable factors between IOP and anterior aqueous flare after K-115 treatment

**Fig 3:**
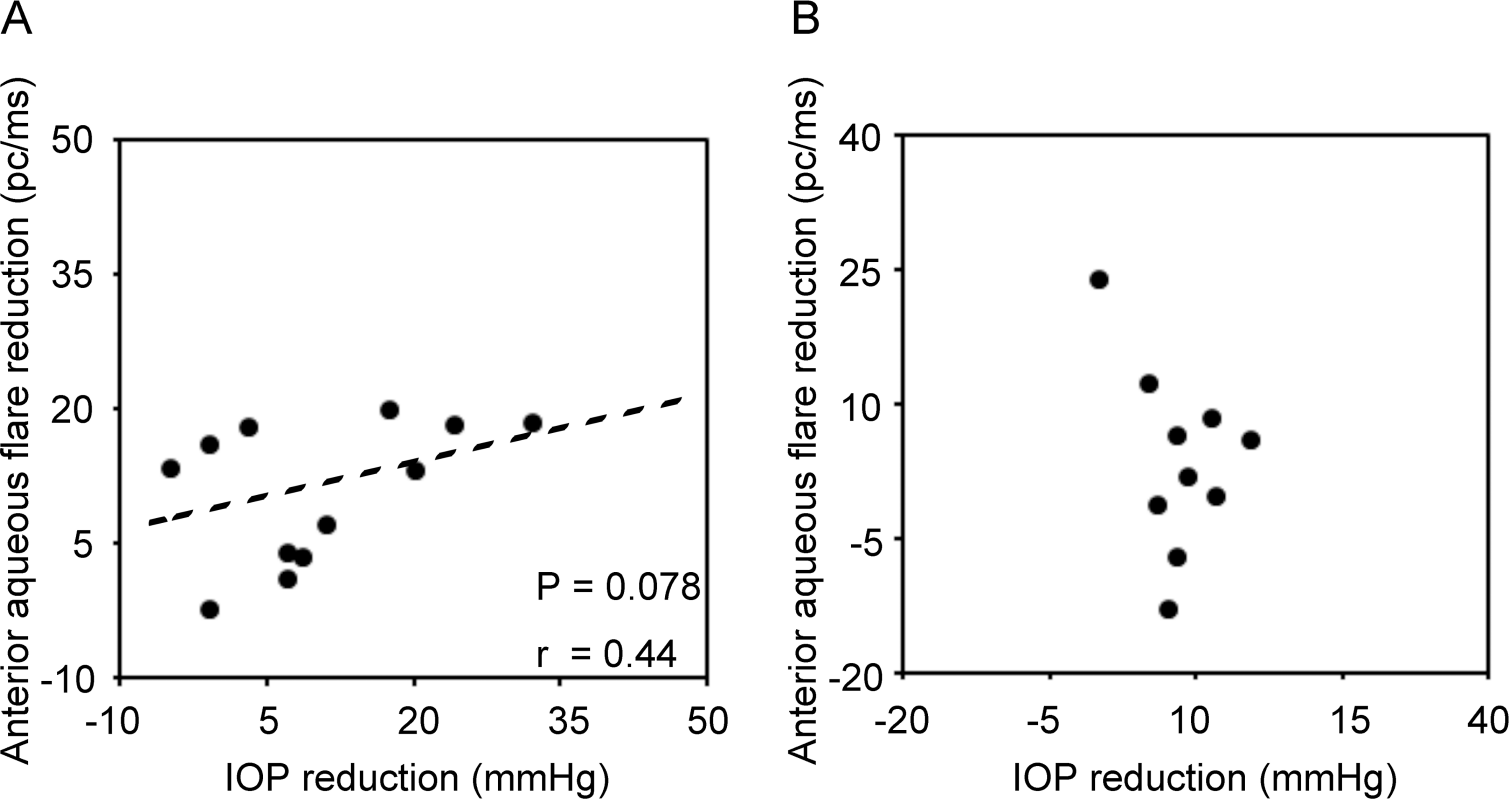
Correlations between intraocular pressure and anterior aqueous flare in the inflammation- and corticosteroid-induced ocular hypertension groups. **(A)** There was a tendency of correlation between IOP reduction and anterior flare decrease in the inflammation group. **(B)** There was no significant correlation between IOP reduction and anterior flare change in the corticosteroid group.

We evaluated the correlation between IOP reduction and anterior aqueous flare change. In the inflammation group, there was a tendency of correlation between IOP reduction and anterior aqueous flare decrease (P = 0.078; r = 0.44), but not in the corticosteroid group (Fig 3).

The results of the multiple linear regression analysis are shown in Table 2.

**Table 2:** Factors affecting changes in IOP and aqueous flare.

		IOP		Flare	
		P	β	P	β
Inflammation/corticosteroid		0.95		0.24	
Age		0.51		0.24	
Sex		0.57		0.45	
Granulomatous/non-granulomatous		0.93		0.86	
Prescribed anti-glaucoma agents					
	Prostaglandin analogues	0.23		0.65	
	β-blockers	0.68		0.21	
	Carbon anhydrase inhibitors	0.94		0.63	
	α2-agonists	0.64		0.03	–18.30
Flare at the initiation		0.45		0.21	
IOP at the initiation		<0.001	1.40	0.02	0.56

IOP: intraocular pressure, Flare: anterior aqueous flare

IOP reduction was positively correlated with IOP at the initiation (P < 0.001; β = 1.40), and decrease of anterior aqueous flare was negatively correlated with combination treatment with α2-agonists (P = 0.028; β = −18.3) and positively correlated with anterior aqueous flare at the initiation (P = 0.019; β = 0.056).

## Discussion

K-115 was developed in Japan as an anti-glaucoma agent to decrease IOP, and has suspected neuroprotective properties [5, 7]. A previous study of eyes with uveitis-associated OHT and glaucoma showed that K-115 decreases IOP associated with anterior uveitis and does not exacerbate ocular inflammation [11]. However, their study only included anterior uveitis–associated OHT, and the cases were not classified into inflammation- and corticosteroid-induced OHT. In the present study, we evaluated the efficacy and safety of K-115 on OHT caused by various types of uveitis and compared the efficacy of this drug between inflammation- and corticosteroid-induced OHT. Our results indicated that K-115 decreased IOP in the eyes of both groups and that anterior flare was also decreased in eyes with inflammation-induced OHT, but not in eyes with corticosteroid-induced OHT.

In eyes with uveitis, the intertrabecular spaces are obstructed by inflammatory cells, proteins and fibrin [12, 13]. The trabecular endothelium has the capacity to phagocytose cells and debris obstructing the outflow channels [14]; excessive phagocytosis results in cell migration away from the trabecular meshwork [15], as well as cell autolysis [16]. With decreased trabecular endothelial cell density, there is a reduction in trabecular function, resulting in elevated IOP [17].

The ROCK pathway underlies non-specific inflammation in neurons, and ROCK inhibitors suppress advanced glycation end product–mediated activation and the induction of oxidative stress by downregulating inducible nitric oxide synthase, cyclooxygenase-2, NLRP3, as well as nuclear translocation of nuclear factor kappa B p65 [18]. In addition, the RhoA/ROCK pathway has been shown to suppress IL-17 and IL-21 production by T cells via both non-selective and selective approaches [19]. It is well known that inflammation itself could elevate IOP in uveitis patients [12, 13]. Therefore, it is possible that IOP was also reduced by alleviated inflammation in eyes of inflammation-induced OHT group treated with corticosteroids in addition to K-115. Previous report demonstrated that K-115 decreased IOP by 3.7 mmHg in eyes with primary open angle glaucoma or OHT without uveitis [20]. In the present study, K-115 decreased IOP by 8.5 mmHg in the inflammation-induced OHT in eyes with uveitis. In addition, significant IOP and anterior flare reductions were observed in inflammation-induced OHT. Furthermore, a tendency of positive correlation between decreases in inflammation and IOP was observed in inflammation-induced OHT. It is considered that IOP reduction by K-115 in the inflammation-induced OHT was promoted by anti-inflammatory therapy. These reductions and correlation would be reasonable results by the mechanism of inflammation-induced OHT and the ROCK/Rho pathway.

K-115 might have a powerful effect in corticosteroid-induced OHT because ROCK inhibitors alter the cellular characteristics of trabecular meshwork affected by systemic or topical corticosteroids. Corticosteroids increase cell stiffness in the trabecular meshwork [21], thus increasing the barrier function of the trabecular meshwork cell monolayer by affecting cell-cell junctions [22]; the tight junction–associated protein ZO-1 and the gap junction protein GJA1 are also increased in glaucomatous trabecular meshwork cells [23]. In the present study, the mean IOP in the corticosteroid group decreased significantly, and the amount of reduction was correlated with the IOP at the initiation. Thus, we believe that K-115 is the appropriate medication for corticosteroid-induced OHT in uveitic eyes.

In the present study, multiple linear regression analysis indicated a negative correlation between anterior flare reduction and combination therapy of K-115 and α2-agonists, which suggests that α2-agonists with or without K-115 possibly increase anterior inflammation in uveitis with OHT. Previous reports have proposed that adrenergic agonists decrease the volume of conjunctival cells and widen the space between cells, allowing greater access of pro-inflammatory agents and continued topical administration of α2-agonists in eyes with allergic reactions may predispose to the development of uveitis [24]. Histological findings of conjunctivitis and anterior uveitis induced by α2-agonists have also been reported [25]. Patients with dark irises may be more susceptible to inflammation because α2-agonists reach a higher concentration in pigmented tissues [26]. These results, in addition to our findings, suggest that α2-agonists may not be an appropriate medication for OHT associated with uveitis and that it may affect aqueous flare outcomes. Additional research comparing the safety of α2-agonists with or without a ROCK inhibitor in cases of aqueous flare for uveitis-associated OHT is warranted to estimate the efficacy of α2-agonists.

Four limitations of this study are the small number of patients, the inclusion of both granulomatous and non-granulomatous uveitis, the single-centre study design and the no randomisation. K-115 may have different effects on eyes with granulomatous and non-granulomatous uveitis because of the differences in their pathologies. Further research with more cases is needed to compare the differences in the efficacy and safety of K-115 when used in c combination with other anti-glaucoma agents.

## Conclusion

Our results demonstrated that K-115 is effective in lowering IOP and is safe and well-tolerated in eyes with uveitis-associated OHT, including both inflammation- and corticosteroid-induced OHT. K-115 may suppress the anterior inflammation in eyes with inflammation-induced OHT and may have stronger effects in eyes with corticosteroid-induced OHT. Our findings show that K-115 is an appropriate medication for uveitis-associated OHT.

## Supporting information

S1 Table All patients date included in the study. (XLSX)
